# The Transcription Factor FRA-1/AP-1 Controls Lipocalin-2 Expression and Inflammation in Sepsis Model

**DOI:** 10.3389/fimmu.2021.701675

**Published:** 2021-10-12

**Authors:** Shan Cao, Anne Schnelzer, Nicole Hannemann, Georg Schett, Didier Soulat, Aline Bozec

**Affiliations:** ^1^ Department of Internal Medicine 3, Rheumatology and Immunology, Universitätsklinikum Erlangen, Friedrich-Alexander-Universität (FAU) Erlangen-Nürnberg, Erlangen, Germany; ^2^ Institute of Regenerative Medicine and Biotherapies (IRMB), University of Montpellier, INSERM U1183, Montpellier, France; ^3^ Mikrobiologisches Institut – Klinische Mikrobiologie, Immunologie und Hygiene, Universitätsklinikum Erlangen, Friedrich-Alexander-Universität (FAU) Erlangen-Nürnberg, Erlangen, Germany

**Keywords:** sepsis, transcription factor, AP-1/Fra-1, lipocalin-2, macrophage, endotoxin tolerance

## Abstract

Sepsis is a life-threatening condition characterized by excessive inflammation in its early phase. This is followed by an aberrant resolution phase associated to a prolonged period of immune suppression that can ultimately lead to multiple organ dysfunctions. This immunosuppression can be mediated by the functional reprogramming of gene transcription in monocytes/macrophages in response to prolonged lipopolysaccharide (LPS) exposure. Surprisingly, there is no report on the role of AP-1 transcription factors in this reprogramming process. Herein, we used the endotoxin tolerance model on murine bone marrow-derived macrophages in which tolerant cells stimulated twice with LPS were compared to naïve cells stimulated once. Out of all AP-1 transcription factors tested, *Fosl1* gene stood out because of its unique regulation in tolerized cells. Moreover, we could correlate FRA-1 expression to the expression of an essential anti-inflammatory molecule involved in sepsis response, Lipocalin 2 aka NGAL. Identical results were obtained in human PBMC following the endotoxin tolerance model. When using FRA-1 deficient macrophages, we could confirm that FRA-1 regulates NGAL expression during the tolerant state. Interestingly, ChIP-seq and ChIP-qPCR revealed the binding of FRA-1 on *Lcn2* promoter after LPS stimulation in these cells. Finally, we used an *in vivo* septic model of consecutive injection of LPS, in which the second stimulation is performed before the resolution of inflammation, in wild type and FRA-1 deficient mice. NGAL secretion was elevated in lung, spleen and serum of wild type tolerant mice, whereas it was significantly lower in tolerant FRA-1 deficient mice. Moreover, an increased inflammatory state likely dependent of the low level of NGAL was observed in these FRA-1 deficient mice. This was characterized by an increase of neutrophil infiltration in lung and an increase of apoptotic follicular cells in spleen. This suggests that FRA-1 expression supports resolution of inflammation in this model. Collectively, our data indicate that FRA-1 is involved in myeloid cell tolerance responses by mediating the functional reprogramming of *Lcn2* transcription in response to prolonged LPS exposure. In conclusion, FRA-1 may have a protective role in the tolerance response of sepsis through the regulation of NGAL, leading to resolution of inflammation.

## Introduction

Sepsis is a life-threatening condition, which represents the leading cause of death in intensive care units (ICUs) worldwide (1). In the last decades, numerous treatments to ameliorate sepsis outcome have failed in clinical trials. There is therefore a strong need to better understand the underlying sepsis mechanism to develop new therapeutic strategies (2). Sepsis is caused by a dysregulated host response to infection that consists of two dynamic phases. The initial and uncontrolled systemic inflammatory response (SIRS) is followed by an unchecked compensatory anti-inflammatory response (CARS). This latter phase often fails to adequately resolve the systemic inflammation and predisposes to immune dysfunction. The failure of resolution process can lead to collateral tissue destruction and multiple organ dysfunction often lethal (3).

The most prominent feature of sepsis-induced immunosuppression is the diminished capacity of innate immune cells, especially monocytes and macrophages, to release pro-inflammatory cytokines in response to pathogen associated molecular patterns (PAMP) (3). Ultimately, these deactivated macrophages adopt an immunosuppressive phenotype making them unresponsive to secondary infections, but prone to resolve inflammation and regenerate tissue. In the case of sepsis, this tolerization process can be studied in the context of endotoxin tolerance induced by consecutive LPS stimulations (4).

Two classes of genes have been categorized in tolerant macrophages in previous studies: genes not inducible described as ‘tolerizable’ genes and genes still inducible named ‘non-tolerizable’ genes (5). Interleukin-6 (*Il6*), known as a classical pro-inflammatory cytokine in sepsis, is a typical tolerizable gene. Despite being a TLR inducible gene, the induction of IL-6 expression by macrophages was abolished after a second LPS stimulation. On the other side, non-tolerizable genes such as lipocalin-2 (*Lcn2*) were found to be significantly elevated in tolerant macrophages (5). Also known as neutrophil gelatinase-associated lipocalin, NGAL is a mammalian protein expressed by myeloid and epithelial cells in response to TLR activation during infections. NGAL plays an anti-microbial role by inhibiting bacterial siderophores and therefore depriving bacteria of iron (6). In parallel to this anti-microbial function, NGAL expression is also important for the immune response. In a LPS-induced sepsis model, *Lcn2* knockout mice showed a dysregulated iron metabolism that resulted in an increased apoptosis of leukocytes, an increased release of pro-inflammatory cytokines and a higher mortality (7). Similarly, NGAL has been shown to induce the production of the anti-inflammatory cytokine IL-10 in macrophages during *Streptococcus pneumoniae* infection, making NGAL not only a marker of macrophages deactivation but also a deactivator of macrophages (8). Interestingly, a feed forward regulation between IL-10 and NGAL has been suggested in another sepsis model, in which NGAL expression correlates with IL-10 level and a better outcome after polymicrobial sepsis (9). Collectively, these researches imply that NGAL plays a role in controlling inflammation and thereby contributes to the resolution of severe inflammation (7). However, its transcriptional regulation, especially in the tolerant cells remains to be defined since regulating the expression of NGAL may provide novel therapy for the resolution of sepsis.

Recent investigations have focused on the mechanisms underlying the functional reprogramming of the gene transcription in macrophages in response to prolonged LPS exposure. Up-regulation of hypoxia-inducible factor-α (HIF-1α) and subsequent elevation of the negative regulator of TLR signaling IRAKM have been involved in the establishment of an endotoxin-tolerant state in monocytes which interestingly possess anti-bacterial and wound-healing properties (10). At the transcriptional level, the cyclin-dependent kinase inhibitor p21 has been shown to tune NF-κB pathway that controls part of the macrophages reprogramming happening during endotoxin tolerance. A high level of p21 favors p50-p50 over p65-p50 for binding to NF-κB sites on DNA, resulting in lower IFN-β production and a hypo-responsive phenotype of macrophage (11). Surprisingly, no data has been reported on the role of AP-1 transcription factors in this reprogramming process. AP-1 family is mainly composed of c-Fos and c-Jun related transcription factors. Depending on stimuli and cell type, they can be activated by phosphorylation or methylation, promptly assembled into homodimers or heterodimers through their leucine-zipper domain and bound to AP-1 sites on target gene promoter, resulting in the promotion or inhibition of gene expression (12).

In the present work, we studied AP-1 transcription factors role using the endotoxin tolerance model in both murine bone marrow-derived macrophages (BMDM) and human peripheral blood mononuclear cell (PBMC). We found that the expression of FRA-1 and NGAL were positively correlated in response to LPS, both in tolerant murine and human cells *in vitro*. With the help of FRA-1 deficient BMDM, we showed that FRA-1 positively regulates NGAL expression especially in the tolerant state. Using ChIP-seq and ChIP-qPCR, FRA-1 was shown to bind *Lcn2* promoter, especially in the tolerant state. Finally, *in vivo* septic model using consecutive injection of LPS showed a decreased resolution as well as an increased inflammation in FRA-1 depleted conditions, likely dependent on the low level of NGAL in these mice. Collectively, our results indicate that FRA-1 transcription factor is involved in myeloid cell tolerant responses by mediating the functional reprogramming of *Lcn2* transcription in response to prolonged LPS exposure.

## Methods and Materials

### Animals

Wild type C57Bl/6NCrl mice were purchased from Charles River. The generation of *Fosl1* floxed and *Mx*-Cre mice has been described before (13). Briefly, the *Fosl1* gene knockout in *Fosl1* ^∆Mx^ mice was induced 2 weeks before the experiment by 3 consecutive injections of 250 μg poly(I:C) per mouse every other day. Littermate *Fosl1* ^fl/fl^ mice were used as controls and were treated with poly (I:C) like the deficient mice. Mice were bred and maintained on a 129/B6 mixed background. All experiments were performed with 8-12-week-old mice (both male and female). Animals were kept under standardized conditions. A 12-hour light/12-hour dark cycle was maintained, and standard diet and water were provided ad libitum. All animal experiments were discussed and approved by the University of Friedrich-Alexander-Universität Erlangen-Nürnberg ethics committee and carried out in accordance with protocols approved by the German law.

### Cell Preparation

Bone marrow-derived macrophages (BMDM) were generated from total bone marrow cells flushed out from 8-12 weeks old mice. The total bone marrow cells were then incubated within 10 cm petri dishes in 5% CO_2_ at 37°C in DMEM and GlutaMAX (Gibco, Thermo Fisher Scientific) supplemented with 10% (v/v) FCS (Gibco, Thermo Fisher Scientific), 1% (v/v) penicillin/streptomycin (Gibco, Thermo Fisher Scientific), and 20% (v/v) supernatant from L929 fibroblast cultures [ATCC clone CCL-1] as a source of macrophage colony-stimulating factor (M-CSF) (14). After overnight incubation, the non-adherent cells were then harvested and reseeded into 10 cm petri dishes supplemented with the medium above at the concentration of 5x10^6^ cells/dish. 7 days later, BMDM were carefully harvested, counted and replated into 12-well plates at the concentration of 1x10^6^ cells/well in RPMI-1640 (Gibco, Thermo Fisher Scientific) supplemented with 10% (v/v) FCS (Gibco, Thermo Fisher Scientific) and 1% (v/v) penicillin/streptomycin (Gibco, Thermo Fisher Scientific). After overnight incubation, the adherent cells were stimulated with 10 ng/mL LPS (Naïve) or stimulated with 100 ng/mL LPS for 24 h, washed twice with warm PBS and stimulated again with 10 ng/mL LPS (Tolerant) as described in previous study (5).

Human peripheral blood mononuclear cells (PBMCs) were isolated from EDTA-blood of normal, healthy donors, using a Ficoll gradient (Lymphoflot, Bio-Rad). PBMCs were then incubated overnight in RPMI-1640 (Gibco, Thermo Fisher Scientific) supplemented with 10% (v/v) FCS (Gibco, Thermo Fisher Scientific) and 1% (v/v) penicillin/streptomycin (Gibco, Thermo Fisher Scientific) and adherent cells were stimulated with once or twice LPS as described above.

### Isolation of CD11b^+^ Cells

The single cell suspensions from the organs were created by using gentleMACS dissociation tubes (Miltenyi Biotec). CD11b^+^ cells were then isolated *via* CD11b microbeads (Miltenyi Biotec) under the manufacturer’s instructions.

### Real-Time PCR Analysis

A standard phenol-chloroform extraction was performed with Trizol reagent to isolate total RNA from cells. cDNA was synthesized from 1 μg of total RNA with a High-Capacity cDNA Reverse Transcription Kit (ThermoFisher). Real-time PCR analysis was then performed and analysed with a SYBR label (SYBR Select Mastermix, Applied Biosystems) in QuantStudio™ 6 Flex Real-Time PCR System. The real-time PCR primer sequences are listed in **Supplementary Table 1**. The relative expression of the gene of interest (GOI) was calculated with the ΔΔCt method using β-actin (*Actb*) expression as normalizer (norm) and the not-stimulated naïve control as calibrator: relative expression = 2^-ΔΔCt^ where ΔΔCt = (Ct_GOI_ - Ct_norm_)_unknown_ - (Ct_GOI_ - Ct_norm_)_calibrator_.

### ELISA

The IL-6 and NGAL concentrations in the relevant supernatant or serum were quantified with the Mouse IL-6 DuoSet ELISA Kit and Mouse Lcn2 DuoSet ELISA Kit following the manufacturer’s instructions (R&D Systems).

### Western Blot Analysis

Proteins from BMDM stimulated once or twice with LPS were collected at 0, 0.5, 1, 2 or 4 h with RIPA buffer supplied with protease inhibitors (Roche, cOmplete™ ULTRA Tablets, 05892970001). The protein extracts were then separated by SDS-PAGE electrophoresis and semi-dry transferred to a PVDF membrane. The membrane was incubated with antibodies against FRA-1 (sc-605, Santa-Cruz, 1: 200) and GAPDH (#2118, Cell Signalling, 1:1000) overnight at 4°C.

### Chromatin Immunoprecipitation-seq and ChIP-qPCR

The ChIP-seq results have been deposited online in the GEO datatabase (GSE178865) along with our previous publication (13). The raw ChIP-seq data were re-analyzed with GALAXY (15) to explore the binding of FRA-1 on the promoter sites of *Il6* and *Lcn2*, with or without stimulation with LPS. In brief, the data sets were firstly trimmed by Trimmomatic to remove adapters and trim reads to improve the overall quality. The sequenced reads were mapped using Bowtie2. The peak-calling step was then performed by MACS2 to identify significantly enriched loci in the genome. Finally, the output files were visualized with the help of Integrated Genome Browser (16).

For further promoter analysis, the 4.000 bp nucleotide sequences upstream of the transcription start sequence (TSS) of *Il6* and *Lcn2* genes were identified thanks to the NCBI Gene database. The putative AP-1 binding sites on *Il6* and *Lcn2* promoters were predicted *via* an online tool TFBIND (17) and relative primers to detect each site were designed accordingly.

ChIP-qPCR on peritoneal macrophages and BMDM was performed with the ChIP-IT High Sensitivity^®^ (HS) Kit (Active Motif) following the manufacturer’s instructions. Briefly, thioglycollate-elicited macrophages were generated by injecting mice i.p. with 2.5 mL of 4% (w/v) Brewer’s thioglycolate medium (Sigma-Aldrich). FRA-1 deficient mice and littermates were sacrificed 72 hours after injection. Peritoneal cavity cells were harvested by lavage, and cells were washed and plated in RPMI-1640 (Gibco, Thermo Fisher Scientific) supplemented with 10% (v/v) FCS (Gibco, Thermo Fisher Scientific) and 1% (v/v) penicillin/streptomycin (Gibco, Thermo Fisher Scientific). BMDM were prepared as described above (Cell Preparation). After overnight incubation, adherent cells were stimulated with 10 ng/mL LPS for 1h (Naïve) or stimulated with 100 ng/mL LPS for 24 h, washed twice with warm PBS and stimulated again with 10 ng/mL LPS for 1h (Tolerant). The cells were fixed with 1% formaldehyde for 15 min to cross-link the proteins to the DNA and then washed for 3 times. The pellet was re-suspended in ChIP lysis buffer and lysate was sonicated to shear DNA to an average fragment size of 200–1000 bp. 30 µg chromatins were incubated with antibodies against FRA-1 (sc-605 from Santa-Cruz for peritoneal macrophages, since the production of this antibody has been discontinued by the company, we used PA5-81175 from Invitrogen for BMDM, 5µg/sample) or Normal Rabbit IgG (#2729, Cell Signalling, 5µg/sample) overnight at 4°C. On the second day, samples were incubated with protein A/G beads for 3 hours. DNA was then eluted, de-crosslinked overnight and purified. 25 µl starting chromatins without incubation with antibodies were also de-crosslinked and purified, named as Input. The enrichment of each binding site was evaluated by ChIP-qPCR. The data was normalized and calculated with Percent Input Method: Adjust Input=Ct_Input_-log2(V_Chromatin_/V_Input_), V means volume. % Input= 100*2^(Adjusted Input - Ct Chromatin)^. The ChIP-qPCR primer sequences are listed in **Supplementary Table 1**.

### *In Vivo* Endotoxin Tolerance Model

The *in vivo* endotoxin tolerance model was established by consecutive i.p. injection of LPS. Briefly, mice were rendered tolerant to LPS by an initial injection of 25 µg of LPS and stimulated 3 days later with another similar dose of LPS for 2 h. The naïve group injected only once with LPS and sacrificed 2h after (**Figure 5A**). PBS-injected mice served as control.

### Histological Analysis

Mice were sacrificed with CO_2_ at indicated time point under respective treatment. The organs were then harvested and fixed in 4% formaldehyde for 12 h. Then, the samples were embedded in paraffin, cut to 1 μm thickness and mounted onto glass slides. For HE staining, the slides were stained with Hematoxylin and Eosin as common procedure. To quantify the LPS-induced changes in the spleen, the follicular areas were measured with ImageJ in 4-fold magnified images. For the evaluation of pulmonary inflammation in response to LPS, the categories “cellular infiltration” and “alveolar wall thickening” were used as described in previous study (18). Briefly, for cellular infiltration, 0 means no immune cell infiltration, 1 means moderate infiltration and 2 means massive infiltration. For alveolar wall thickening, 0 is normal, 1 represents thickening fewer than 50% while 2 is over 50%. Four representative images in 20-fold magnification per slide were analyzed and the respective values for each sample were summed up and then compared among diverse treatments.

### TUNEL Staining

TUNEL staining of the spleen slides were performed with the In Situ Cell Death Detection-Kit (POD) following the manufacturer’s instructions. Four representative images in 20-fold magnification per slide were analyzed and the apoptotic splenocytes within the follicular area were counted and compared among diverse treatments.

### Data Analysis

GraphPad Prism version 8.0 (GraphPad Software, San Diego, CA) was used for statistical treatment. Experimental data was shown as mean ± s.e.m. Two-tailed unpaired student’s t-test for two groups or ANOVA with multiple comparisons test were used as indicated in respective experiments. For kinetic expression of genes and proteins, repeated measures two-way ANOVA (two-way RM ANOVA) was used in which the time factors are matched, to account for the nestedness of the data.

## Results

### *Fosl1* Expression Is Altered After Induction of Endotoxin Tolerance in Murine Macrophages

**Figure 1:**
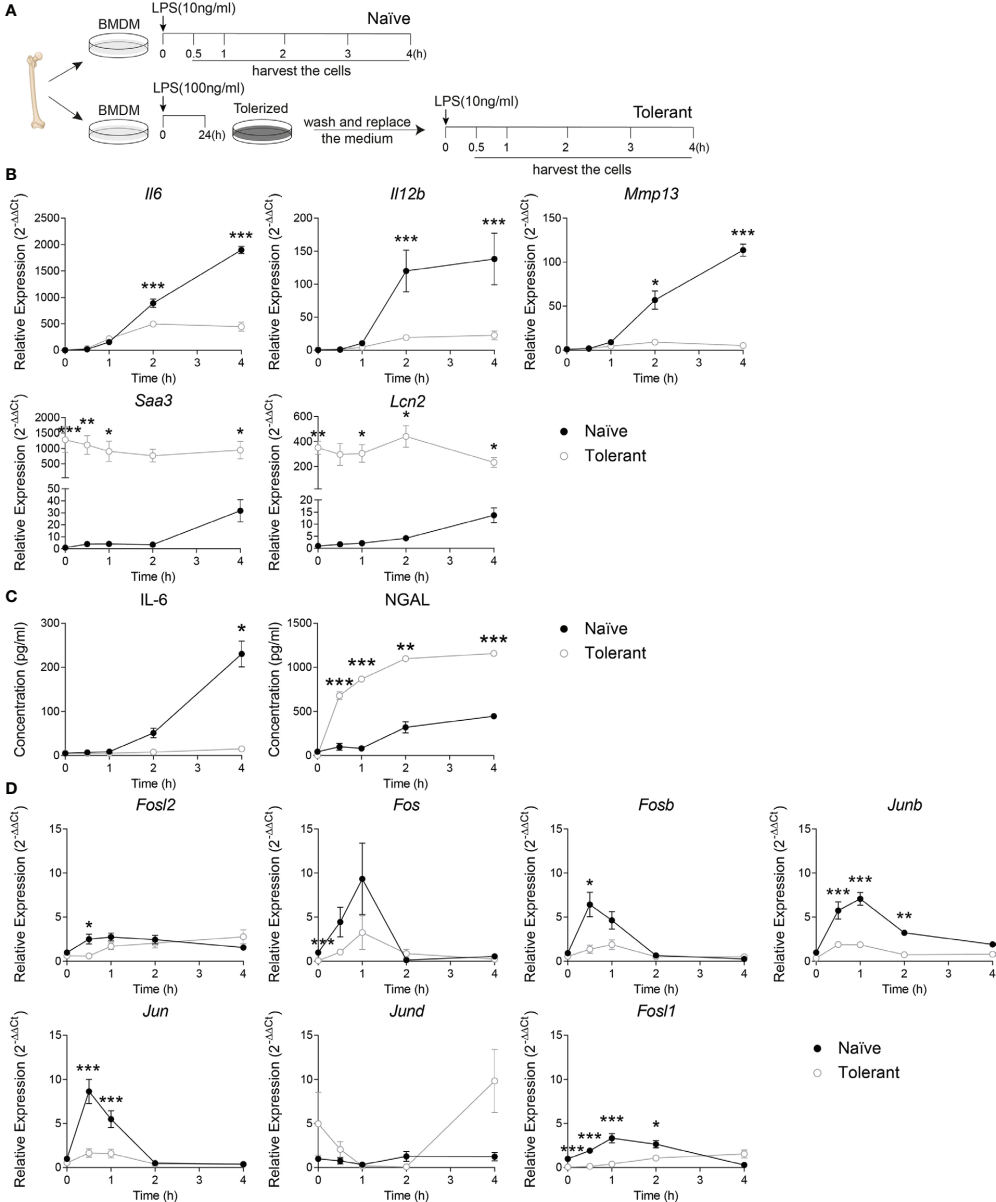
*Fosl1* expression is altered after induction of endotoxin tolerance in murine macrophages. **(A)** Experimental scheme of the induction of endotoxin tolerance in wild type murine BMDM. A kinetic analysis of genes and proteins expression was performed at the indicated time point in macrophages harvested following one (Naïve) or two (Tolerant) LPS stimulations. **(B)** Quantitative RT-PCR analysis of the expression of tolerizable genes (*Il6, Il12b Mmp13*) and non-tolerizable genes (*Saa3, Lcn2*), normalized to the expression of *Actb*. **(C)** Quantification of IL-6 and NGAL protein secretion in cell culture supernatant. **(D)** Quantitative RT-PCR analysis of AP-1 transcription factor genes, normalized to the expression of *Actb*. Data are shown as mean ± s.e.m. *p < 0.05; **p < 0.01; ***p < 0.001 by two-way RM ANOVA with Tukey’s multiple comparisons test. The data shown are from one experiment representative of three independent biological replicates.

To evaluate the AP-1 expression within the two phases of sepsis, we first set up the endotoxin tolerance model in wild type murine bone marrow-derived macrophages (BMDM) by consecutive stimulation of LPS (**Figure 1A**) (5). The adherent BMDM were stimulated with 10 ng/mL LPS (Naïve) or stimulated with 100 ng/mL LPS for 24 h, washed twice with warm PBS and stimulated again with 10 ng/ml LPS (Tolerant). The cells were then harvested at 0, 0.5, 1, 2 or 4 h after the stimulation. The validity of our experimental model was confirmed by analyzing the kinetic expression of genes previously known as tolerizable or non-tolerizable (5) at the indicated time point in naïve and tolerant macrophages. The tolerizable genes (*Il6, Il12b* and *Mmp13*) were significantly up-regulated in the naïve cells, while significantly diminished in the tolerant cells compared to the naïve one. Meanwhile, the non-tolerizable genes (*Saa3, Lcn2*) were only weakly induced by LPS in naïve cells but showed a strong and sustained expression in stimulated tolerant cells (**Figure 1B**). The analysis of the protein secretion for IL-6 and NGAL (neutrophil gelatinase-associated lipocalin, protein name of *Lcn2*) in the supernatant of murine BMDM further confirmed the mRNA results (**Figure 1C**). IL-6 cytokine levels in the supernatant were significantly elevated after the first LPS stimulation, and abolished after the second stimulation, while the NGAL protein expression was still increased in the tolerant state. After this confirmation, we studied the expression kinetic of AP-1 transcription factor genes in murine BMDM in both naïve and tolerant state. The expression of *Fosl2, Fos, Fosb, Junb* and *Jun* peaked shortly (0.5-1h) after LPS stimulation in naïve cells, whereas the induction of these genes was strongly diminished in tolerant cells (**Figure 1D**). In this homogenous family picture, two AP-1 members stood out: *Jund* was unresponsive to LPS stimulation and was therefore not considered further, whereas *Fosl1* response was more interesting. In naïve cells, *Fosl1* responded alike the other AP-1 transcription factors. However, in tolerant cells, *Fosl1* did not show the small expression peak observed for the other AP-1 members but an initial level of expression dramatically reduced (6% of the unstimulated naïve cells) that slowly and steadily increased after LPS re-stimulation. Therefore, our results in wild type BMDM suggested that the induction of AP-1 gene expression by LPS stimulation in naïve cells was strongly reduced in tolerant cells, and Fra-1 expression seemed unique compared to the other members.

### *Fosl1* Expression Is Altered After Induction of Endotoxin Tolerance in PBMC

**Figure 2:**
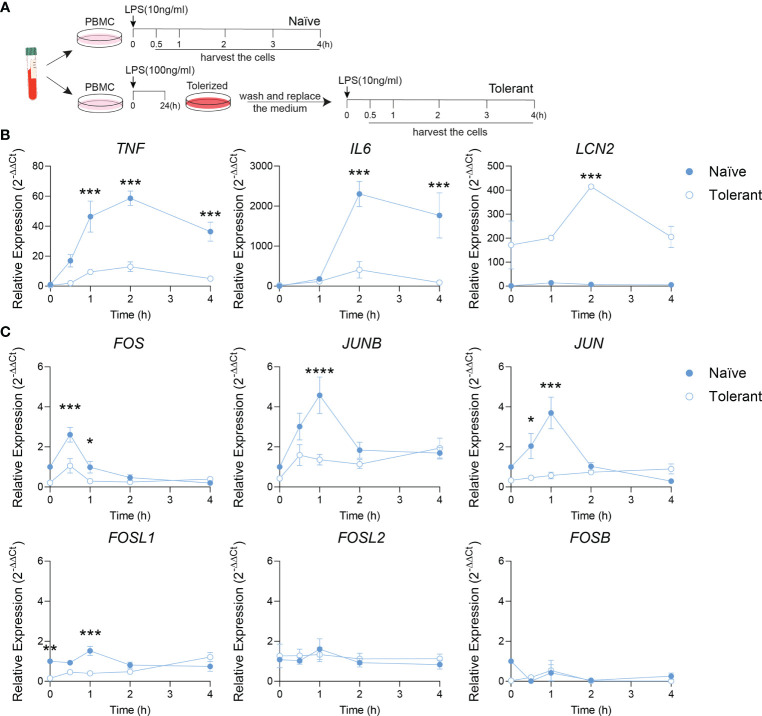
*Fosl1* expression is altered after induction of endotoxin tolerance in human peripheral blood mononuclear cells. **(A)** Experimental scheme of the induction of endotoxin tolerance response in human PBMC. A kinetic analyses of gene expression was performed at the indicated time point in PBMC harvested following one (Naïve) or two (Tolerant) LPS stimulations. **(B)** Quantitative RT-PCR analysis of tolerizable genes (*TNF, IL6*) and non-tolerizable genes (*LCN2*), normalized to the expression of *Actb*. **(C)** Quantitative RT-PCR analysis of AP-1 transcription factor genes, normalized to the expression of *Actb*. Data are shown as mean ± s.e.m. *p < 0.05; **p < 0.01; ***p < 0.001; ****p < 0.0001 by two-way RM ANOVA with Tukey’s multiple comparisons test. The data shown are from one experiment representative of three independent biological replicates.

To verify whether our findings on AP-1 response in tolerant state can be transposed in human, the endotoxin tolerance was studied in isolated human PBMC (**Figure 2A**). Alike in murine cells, the tolerizable genes (*TNF, IL6*) were up-regulated by LPS stimulation in naïve cells, but could not be induced similarly in the tolerant cells. As expected, the expression of *LCN2* was higher in the tolerant state compared to the naïve one (**Figure 2B**). These data demonstrate a response of human PBMC to endotoxin tolerance consistent with the one observed in mouse macrophages. Next, AP-1 gene transcriptions were analyzed. Alike in mouse cells, the peak expression of *FOS, JUNB* and *JUN* observed in naïve cells after 0.5-1 h of LPS stimulation diminished in stimulated tolerant cells. Here again, *FOSL1* expression was initially strongly decreased in tolerant cells and could be gradually increased up to 8-fold (1.21/0.15) by LPS stimulation (**Figure 2C**). Despite that this pattern was reproduced by other AP-1 transcription factors gene like *JUNB* (4.6-fold, 1.94/0.43) and *JUN* (2.7-fold, 0.90/0.33), *FOSL1* was the most LPS responsive AP-1 gene. Therefore, these results established that the data obtained with the mouse macrophages can be confidently transposed to human PBMC.

### Increased Expression of NGAL in Tolerant Macrophages Depends on FRA-1 Expression

**Figure 3:**
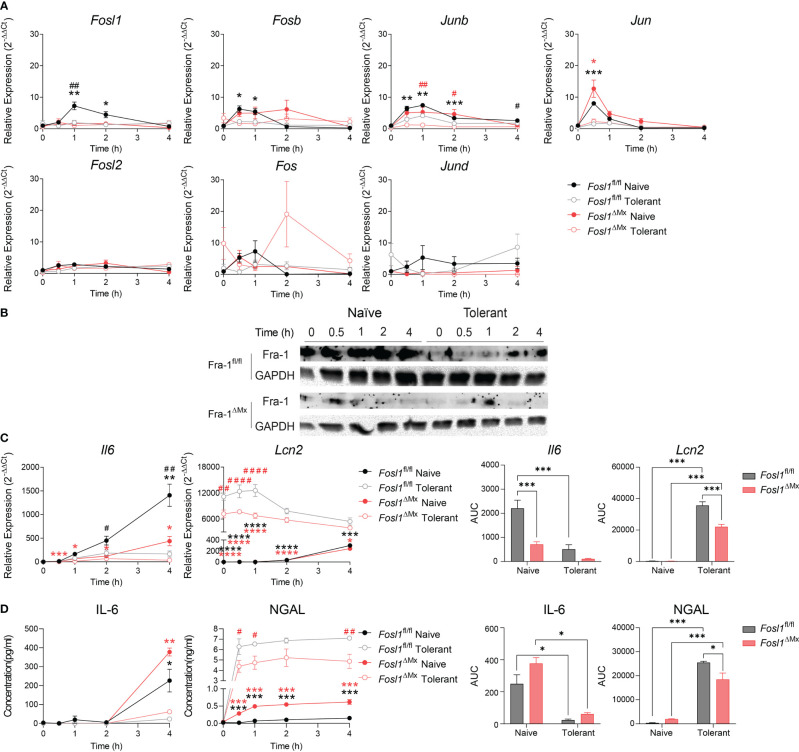
Increased expression of *Lcn2* after induction of endotoxin tolerance is reduced in *Fosl1* knockout murine macrophages. **(A)** Quantitative RT-PCR analysis of AP-1 transcription factor gene expression in littermate control and Fra-1 deficient BMDM after LPS stimulation in both naïve and tolerant state, normalized to the expression of *Actb*. **(B)** Western blot detection of FRA-1 protein after LPS stimulation in littermate control and FRA-1 deficient BMDM in both naïve and tolerant state. GAPDH was used as loading control. **(C)** Quantitative RT-PCR analysis of *Il6* and *Lcn2* in littermate control and FRA-1 deficient BMDM after LPS stimulation in both naïve and tolerant state. Left panels represent the kinetic of gene expression normalized to the expression of *Actb* and right panels represent the area under curve (AUC) for the respective genes. **(D)** IL-6 and NGAL protein levels in the supernatant of wild type and FRA-1 deficient BMDM after LPS stimulation in both naïve and tolerant state. Left panels represent the kinetic of protein secretion and right panels represent the area under curve (AUC) for the respective protein. The data shown are from one experiment representative of three independent biological replicates and they are shown as mean ± s.e.m. *p < 0.05; **p < 0.01; ***p < 0.001; ****p < 0.0001 by two-way RM ANOVA with Tukey’s multiple comparisons test. Black asterisks indicated significant difference between naïve (n=6) versus tolerant (n=9) state in littermate control mice; red asterisk indicated significant difference between naïve (n=3) versus tolerant (n=9) state in FRA-1 deficient mice; ^#^p < 0.05; ^##^p < 0.01; ^####^p < 0.0001 by two-way RM ANOVA with Tukey’s multiple comparisons test. Black hash sign indicated significant difference between FRA-1 deficient and littermate cells in naïve state; red hash sign indicated significant difference between FRA-1 deficient and littermate cells in tolerant state.

Considering the significantly different expression of *Fosl1* in naïve and tolerant state in both murine and human phagocytes, we speculated that FRA-1 could play an important role in regulating the tolerance induced transcriptional reprogramming. To evaluate how FRA-1 influenced the inflammatory response in endotoxin tolerance, inducible FRA-1 deficient mice were generated by crossing *Fosl1* lox mice to *Mx*-Cre mice. After the induction of FRA-1 deficiency by consecutive poly (I: C) injection, BMDM were isolated from the FRA-1 deficient mice and their littermates to study their response to the endotoxin tolerance model *in vitro*. After LPS stimulation, naïve macrophages from littermates induced *Fosl1* expression following a similar pattern as wild type macrophages but to a higher extent. This slightly increased *Fosl1* expression might be due to the heterogeneity of their genetic background. The up-regulation of *Fosl1* by LPS was abolished in the naïve and tolerant FRA-1 deficient BMDM (**Figure 3A**). The mRNA expression profiles of other AP-1 members such as *Fosb, Junb* and *Jun* were not significantly altered by the absence of *Fosl1* and responded similarly to the LPS stimulation (**Figure 3A**). This transcriptional data could be correlated at the translational level: FRA-1 expression after LPS stimulation is reduced in wild type cells after tolerance induction and is strongly diminished in FRA-1 deficient cells (**Figure 3B**).

To determine the influence of FRA-1 on the regulation of the tolerizable and non-tolerizable genes, we next investigated the expression pattern of *Il6* and *Lcn2* in FRA-1 deficient cells. In absence of *Fosl1*, expression of *Il6* mRNA was strongly decreased, especially in naïve state (**Figure 3C**, black hash sign), without significantly altering the early secretion of IL-6 protein (**Figure 3D**). Interestingly, the elevated expression of *Lcn2* in the tolerant cells was significantly reduced in FRA-1 knockout macrophages both at mRNA and protein levels (**Figures 3C, D**). This data suggested that expression of FRA-1 and NGAL are positively correlated, especially in tolerant cells.

### FRA-1 Transcriptionally Regulates *Il6* and *Lcn2* at the Promoter Level

**Figure 4:**
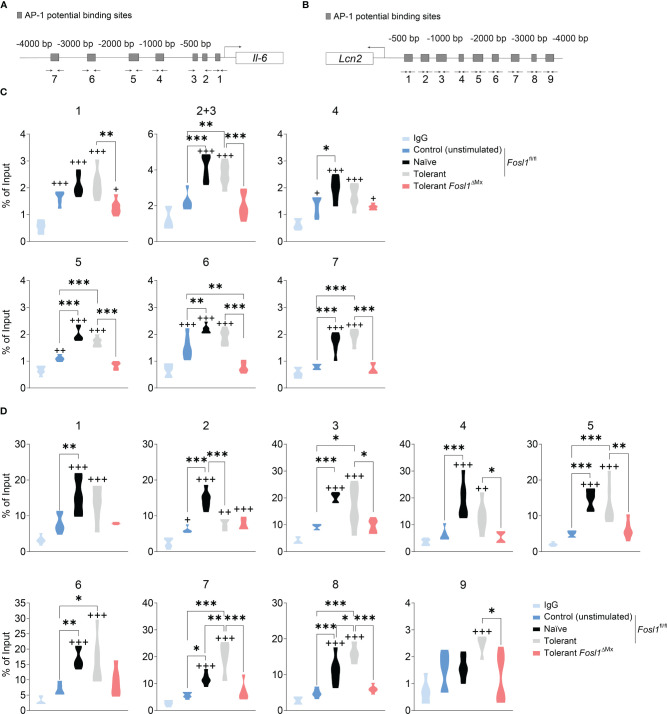
FRA-1 binds stronger to the promoter of *Lcn2* after induction of endotoxin tolerance in murine BMDM. **(A, B)** Prediction of the AP-1 binding sites by the online tool TFBIND and the locations of the primers for each site on *Il6* **(A)** and *Lcn2* **(B)** promoters. **(C, D)** ChIP assays showing the recruitment of the endogenous FRA-1 on the AP-1 binding sequence of *Il6* **(C)** and *Lcn2* **(D)** promoters after one (naïve) or two (tolerant) stimulations with LPS in bone marrow-derived macrophages isolated from *Fosl1* ^ΔMx^ or littermate mice. ^+^p < 0.05; ^++^p < 0.01; ^+++^p < 0.001 by one-way ANOVA with Sidak’s multiple comparisons test. + means compared to the IgG group. *p < 0.05; **p < 0.01; ***p < 0.001 by one-way ANOVA with Sidak’s multiple comparisons test.

Considering the fundamental role of FRA-1 as a transcription factor, we hypothesized that FRA-1 can regulate directly the transcription of *Il6* and/or *Lcn2* in the context of endotoxin tolerance. Using our previously published ChIP-seq analyses performed in peritoneal macrophages with or without LPS stimulation (13), we discovered that FRA-1 can bind to the promoter sites of *Il6* and *Lcn2*, especially after LPS stimulation. For further analysis of these promoters, we considered the 4.000 bp upstream of their transcription start (TSS) (gray area depicted in **Supplementary Figures 1A, B**). The putative AP-1 binding sites on *Il6* and *Lcn2* promoters were predicted *in silico* (**Figures 4A, B**) (17). To confirm this analysis, ChIP-qPCR were then performed with anti-FRA-1 antibody in BMDM from FRA-1 deficient mice and littermates under naïve or tolerant conditioning and compared to not stimulated cells. In this experiment, *Fosl1* ^∆Mx^ BMDMs expressing minimal amount of FRA-1 were used as control for the anti-FRA-1 antibody specificity. ChIP-seq data were partly confirmed by our new ChIP-qPCR experiment: FRA-1 could be detected on the promoter of *Il6* at the loci 1, 4, 5 and 6 in all conditions including non-stimulated cells. Upon LPS stimulation, naïve cells showed an increase of FRA-1 binding on most of the *Il6* promoter locus (all but 1). A similar pattern was observed upon re-stimulation of tolerant cells and diminished in FRA-1 deficient tolerant cells (**Figure 4C**). Interestingly, FRA-1 could not be detected on most of the loci of *Lcn2* promoter in resting cells. However, LPS stimulation strongly mobilized FRA-1 loci 1 to 8 on this promoter in naive cells. Moreover, this binding could be increased for loci 7 and 8 in tolerant cells (**Figure 4D**). This FRA-1 binding pattern on *Il6* and *Lcn2* promoter was confirmed by performing a similar analysis in peritoneal macrophages (**Supplementary Figures 1C, D**). Therefore, our data implies important transcriptional regulation of the *Lcn2* promoter by FRA-1 especially in the tolerant state.

### Increased Inflammation in the Immunosuppressive Phase of Sepsis in *Fosl1* ^∆Mx^ Mice Compared to Littermates

**Figure 5:**
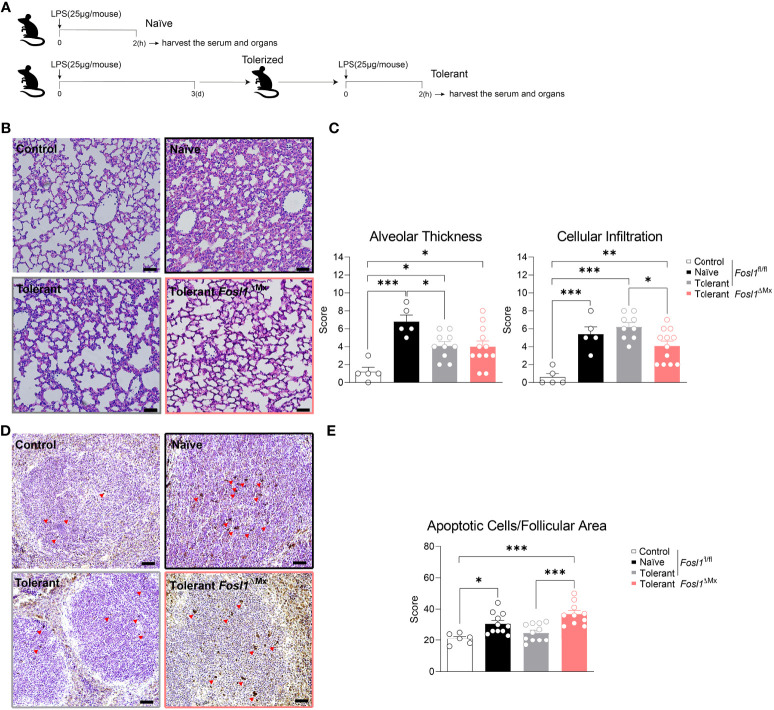
Increased inflammation in the immunosuppressive phase of sepsis is observed in *Fosl1* ^∆Mx^ mice compared to littermates. **(A)** Experimental scheme of the induction of endotoxin tolerance response *in vivo*. **(B, C)** Representative H&E microscopy images and quantification of alveolar thickness and immune cell infiltration in the lung of *Fosl1* ^∆Mx^ and the littermate mice 2 h after once (Naïve) or twice (Tolerant) LPS stimulation. Scale bar = 200μm. **(D, E)** Representative TUNEL staining images and quantification of apoptotic cells adjusted to follicular area in the spleen of *Fosl1* ^∆Mx^ and the littermate mice after once (Naïve) or twice (Tolerant) LPS stimulation. Scale bar = 40μm. Graph points indicate individual mice. Data are shown as mean ± s.e.m. *p < 0.05; **p < 0.01; ***p < 0.001 by one-way ANOVA with Sidak’s multiple comparisons test.

Once established the role of FRA-1 in endotoxin tolerance *in vitro*, we decided to investigate if this finding could be transposed *in vivo* in a model mimicking the induction of endotoxin tolerance observed during sepsis. To do so, we applied consecutive injection of LPS to *Fosl1* ^∆Mx^ and the littermate control mice (**Figure 5A**). Mice were rendered tolerant to LPS by an initial i.p. injection of 25 µg of LPS and stimulated 3 days later with another similar dose of LPS for 2 h. These mice were compared to naive mice injected only once with LPS. PBS-injected mice served as control. Lungs and spleens were then harvested to quantify the inflammation induced by LPS in these organs. *In vivo* injection of LPS in mice is characterized by a recruitment of immune cells to the lung and an increase of the thickness of the alveolar walls. In littermate control mice, LPS injection significantly thickens the lung alveolar walls of naïve mice. Interestingly, this thickening was decreased in tolerant mice despite a similar immune cell infiltration in the lung when compared to naïve mice (**Figure 5B**). This acute inflammatory response could also be observed in *Fosl1* ^∆Mx^ mice but to a lower extend. Tolerant FRA-1 deficient mice showed thickening of their alveolar walls similar to the tolerant littermate control mice but a significantly decreased infiltration of their lung by immune cells (**Figure 5C**). We further investigated the inflammatory conditions in the spleen of these mice. Even though the follicular area of the spleen was significantly increased by LPS injection in the naïve wild type mice, it appears that this criterion went back to normal and became unresponsive to LPS in tolerant wild type mice. No significant difference was observed between the *Fosl1* ^∆Mx^ mice and the littermates in the tolerant state (**Supplementary Figure 2**). Another consequence of systemic inflammatory response induced by LPS is the induction of apoptosis in leukocyte (7). Our TUNEL staining confirmed that LPS injection increased the number of apoptotic cells in the splenic follicular area of naïve mice (control vs naïve, p=0.0224). Interestingly, this induction of apoptosis by LPS was not observed in tolerant mice (control vs tolerant, p=0.7671). This tolerance seems to be dependent on *Fosl1* expression as tolerant FRA-1 deficient mice showed a higher number of apoptotic leukocytes compared to littermate control tolerant mice (tolerant vs tolerant FRA-1 deficient mice, p=0.0005) (**Figures 5D, E**). Altogether, our data suggested that a more severe inflammation as well as a weaker resolution in lung and spleen occurred in tolerant FRA-1 deficient mice compared to their wild type littermates.

### Increased Expression of NGAL in the Immunosuppressive Phase of Sepsis Is Reduced in *Fosl1* ^∆Mx^ Mice

**Figure 6:**
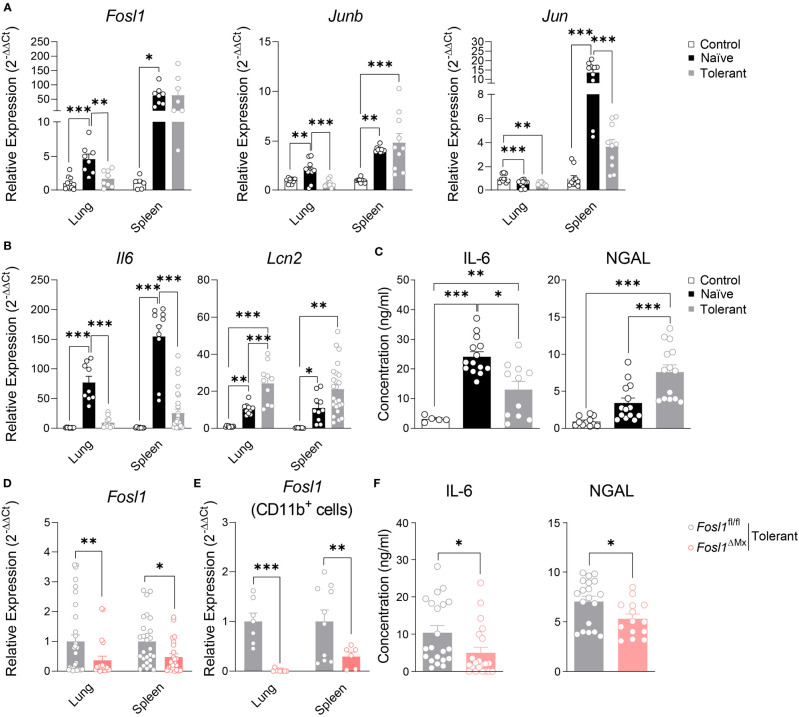
Increased expression of *Lcn2* in the immunosuppressive phase of sepsis is reduced in *Fosl1* ^∆Mx^ mice. **(A, B)** Quantitative RT-PCR analysis of AP-1 transcription factors **(A)** and *Il6* and *lcn2* genes **(B)** in lung and spleen of control, naïve or tolerant wild type mice 2 h after LPS infection. **(C)** IL-6 and NGAL levels in serum of control, naïve or tolerant wild type mice 2 h after LPS infection. **(D, E)** Quantitative RT-PCR analysis of *Fosl1* transcription factor genes in total organ **(D)** or CD11b^+^ cells **(E)** from lungs and spleens of *Fosl1* ^∆Mx^ and the littermate mice in tolerant state, normalized to the expression of *Actb*. **(F)** IL-6 and NGAL levels in serum of *Fosl1* ^∆Mx^ and the littermate mice in tolerant state. Graph points indicate individual mice. Data are shown as mean ± s.e.m. *p < 0.05; **p < 0.01; ***p < 0.001 by one-way ANOVA with Tukey’s multiple comparisons in **(A–C)** and student t test in **(D–F)**.

To understand how AP-1 transcription factors and particularly FRA-1 are involved in our endotoxin tolerance model, we investigated their gene expression profile in murine sepsis model. *Fosl1, Junb* and *Jun* were significantly increased by LPS in both lung and spleen of naïve mice. However, establishment of endotoxin tolerance in wild type mice strongly reduced the increased of *Fosl1* and *Junb* in lung and of *Jun* in spleen by LPS (**Figure 6A**). In the same line, we measured the expression of *Il6* and *Lcn2* as standard tolerizable and non-tolerizable genes respectively, in lung and spleen of the control, naïve and tolerant mice. As expected, *Il6* was strongly induced only in naïve mice whereas *Lcn2* was induced in naive and even more in tolerant mice. For these two genes, protein secretion in the serum fully matched the mRNA data obtained in lung and spleen (**Figures 6B, C**). For the *Fosl1* ^∆Mx^ and littermate control mice, we first quantified the knockout efficiency within the organs and found that *Fosl1* expressions were significantly decreased in lung and spleen in the tolerant state, especially in CD11b^+^ cells (**Figures 6D, E**). Alongside, we could confirm the influence of FRA-1 on the cytokine secretion as FRA-1 deficient mice have a lower level of circulating IL-6 and NGAL (**Figure 6F**). Altogether, our *in vivo* experiments confirm that NGAL expression is controlled by FRA-1 during tolerance induction.

## Discussion

In the present study, we delineated the expressions of the AP-1 transcription factor family *in vitro* with an endotoxin tolerance model applied to murine BMDM and human PBMC and *in vivo* with a murine LPS induced sepsis model. These experiments revealed that the AP-1 transcription factor FRA-1 regulates NGAL expression during the establishment of tolerance in myeloid cells. Interestingly, ChIP-qPCR data further demonstrated a direct regulation on *Lcn2* expression by FRA-1, through its binding on *Lcn2* promoter in tolerant macrophages. This observation could be transposed in the *in vivo* septic model of consecutive injection of LPS. FRA-1 deficient mice showed a decreased resolution of inflammation likely dependent on their low level of circulating NGAL. Thus, our data indicates that FRA-1 is involved in myeloid cell tolerance responses by mediating the functional reprogramming of *Lcn2* transcription in response to prolonged LPS exposure. In addition, FRA-1 may have a protective role in the tolerance response and resolution of inflammation in sepsis through the regulation of NGAL.

Sepsis is a lethal systemic inflammatory response. Due to the high mortality rate and the lack of efficient therapy, the main mechanisms sustaining this disease are still under investigation. Although initially not appreciated, the rapid display of profound immunosuppression in most patients with sepsis is now a well-established phenomenon (4, 19). However, the immunological and molecular mechanism of the immunosuppression is still poorly understood. Here, we used the endotoxin tolerance model as described previously (5) *in vitro* and *in vivo* to mimic the prolonged LPS stimulation that can induce immunosuppression during sepsis. Endotoxin tolerance model is a convenient model for analyzing macrophage hyporesponsiveness. It is induced after exposure of macrophages or mice to a non-lethal low dose of LPS that induces hyperinflammation and then with a second exposure to LPS deactivates the macrophages, resulting in the establishment of immunosuppression (20). With endotoxin tolerance model, we could observe an increase of pro-inflammatory genes upon the first LPS stimulation, and diminished significantly after the second LPS stimulation as shown by Simmie L. Foster and her colleagues (5). We also observed *in vivo* a significantly increased alveolar wall thickness and neutrophil infiltration in the lung and more follicular apoptotic cells in the spleen in the naïve state, while this phenotype was ameliorated in the tolerant state as shown by Frank M. Davis et al. (21), vividly illustrating the whole process of sepsis..

After the tolerance induction, macrophages undergo functional reprogramming and change to an anti-inflammatory phenotype that activates resolution and regeneration programs, promoting a return to tissue homeostasis (11). Scientific advances have highlighted the role of transcriptional regulation in this functional reprogramming process underlying the immunosuppressive phase (10, 11, 22). The AP-1 transcription factor family is involved in numerous processes such as cell proliferation, differentiation and death, depending on the stimulus, the microenvironment and the cell type (12). The wide range of possible combinations and the occurrence of post-translational modifications explain the great functional heterogeneity of AP-1 transcription factors (12, 23). The function of FRA-1 is clearly established in bone and tumor biology (24, 25). There, FRA-1 needs to heterodimerize with another AP-1 transcription to function. This peculiarity makes FRA-1 an interesting transcription factor as its deletion alters the functions of its partners on their shared target genes. Nonetheless, the role of FRA-1 in the context of inflammation, especially concerning *Il6* expression, is still controversial. Mimicking the tumor microenvironment *in vitro*, the 4T1 mammary carcinoma cells promoted *de novo* overexpression of *Fosl1* in RAW264. 7 cells, and the subsequent binding of FRA-1 on the *Il6* promoter and an increase of IL-6 production in response to LPS (26). On the contrary, Morishita et al. reported that IL-6 production after LPS stimulation could be inhibited in RAW264.7 macrophages by overexpressing FRA-1 thanks to a lentiviral vector. This phenotype could be reversed by knockdown of *Fosl1* in this cell line (27). In parallel, no significant difference was observed on *Il6* expression in FRA-1 deficient alveolar macrophages in a LPS triggered acute lung injury model, even though the KO mice exhibited lower mortality rates compared to the control (28). Thus, the function of FRA-1 on IL-6 expression upon inflammation appears to vary depending on the stimulating strategy, the initial intracellular concentration of FRA-1 and the cell type. This multi-factorial context probably influences the non–AP1 transcription factors and the nature of the partner of FRA-1 in the AP-1 heterodimer binding to the *Il6* promoter. This ambivalent function of FRA-1 should rely on opposite regulatory mechanisms. Indeed, FRA-1 has been described as a positive regulator of transcription for genes involved in innate immunity (13) as well as a mediator to repress gene expression in related context (29). In the present study, we could first confirm that *Il6* was a strongly tolerizable gene. In addition, we showed that *Il6* mRNA expression upon LPS stimulation was dependent on the *de novo* expression of Fra-1 in naïve macrophages. If our limited kinetic could not illustrate this FRA-1 dependency for the IL-6 protein secretion *in vitro*, we observed *in vivo* that circulating IL-6 level were decreased in tolerant mice subjected to our sepsis model.

The role of FRA-1 on *Lcn2* expression has yet to be reported. NGAL has been described as a marker of deactivated macrophages and was initially characterized as an anti-microbial protein targeting bacterial siderophore (6, 8). In addition, NGAL supports the establishment of hypoferremia during inflammation to restrict iron availability for the pathogens and thus contributes to the resolution of sepsis (7). Therefore, expression of NGAL promotes microbicidal activities of the immune system and would ultimately contribute to the resolution of sepsis. In our results, the elevated expression of *Lcn2* in the tolerant state was significantly reduced in the *Fosl1* knockout macrophages, at both mRNA and protein level. This correlation between FRA-1 and *Lcn2* expression suggest that this transcription factor offers an additional layer of regulation to the already tightly controlled *Lcn2* promoter. Indeed, transcription factors classically activated under inflammatory conditions such as STAT1 or NF-kB have been previously linked to the regulation of *Lcn2* expression (30, 31). Strikingly, we also observed this correlation *in vivo*. FRA-1 deficient mice subjected to endotoxin tolerance had a significantly decreased level of circulating NGAL. These mice also showed less neutrophil infiltration in the lung and more apoptotic cells in the spleen, implying a higher level of inflammation. Altogether, we propose that FRA-1 have a protective role in response endotoxin induced sepsis through the regulation of NGAL.

Therefore, a better understanding of how AP-1, especially FRA-1, expression and functions in tolerant macrophages can provide valuable insights into the fundamental mechanisms underlying sepsis. Characterizing the regulation of the downstream targets of these transcription factors could open new avenue for the development of therapeutic options against sepsis.

## Data Availability Statement

The datasets presented in this study can be found in online repositories. The names of the repository/repositories and accession number(s) can be found below: https://www.ncbi.nlm.nih.gov/geo/, GSE178865.

## Ethics Statement

The animal study was reviewed and approved by University of Friedrich-Alexander-Universität Erlangen-Nürnberg ethics committee.

## Author Contributions

SC, NH, and AB designed the study. SC, DS, and AB wrote the manuscript. NH contributed to the ChIP-sequencing analysis. SC and AS performed the *in vitro* and *in vivo* experiments. GS supervised the study and edited the manuscript. All authors contributed to the article and approved the submitted version.

## Funding

This work was supported by the Deutsche Forschungsgemeinschaft: BO-3811/5-1; BO-3811/6-1; FOR 2886-TP02; CRC 1181 project A01; SPP2084 µBone; Erlangen-Nuremberg University ELAN-Program P044; Interdisciplinary Centre for Clinical Research (IZKF) grant F1-04; European Research Council (ERC) Synergy Grant 4D Nanoscope and ERC consolidator - ODE.

## Conflict of Interest

The authors declare that the research was conducted in the absence of any commercial or financial relationships that could be construed as a potential conflict of interest.

## Publisher’s Note

All claims expressed in this article are solely those of the authors and do not necessarily represent those of their affiliated organizations, or those of the publisher, the editors and the reviewers. Any product that may be evaluated in this article, or claim that may be made by its manufacturer, is not guaranteed or endorsed by the publisher.

## Supplementary Material

Supplementary Figure 1 FRA-1 binds to the promoters of *Il6* and *Lcn2*. **(A, B)** ChIP-seq data (13) of thioglycollate-elicited macrophages from wild type mice, unstimulated or stimulated with LPS were used to identify FRA-1 target genes. Bindings of FRA-1 on the *Il6* **(A)** and *Lcn2* **(B)** promoter are indicated **(C, D)** ChIP assays showing the recruitment of the endogenous FRA-1 on the AP-1 binding sequence of *Il6* **(C)** and *Lcn2* **(D)** promoters in thioglycollate-elicited macrophages isolated from *Fosl1* ^ΔMx^ or littermate mice after once (naïve) or twice (tolerant) stimulation of LPS. Data are shown as mean ± s.e.m. *p < 0.05; **p < 0.01; ***p < 0.001 by one-way ANOVA with Tukey’s multiple comparisons test.

Supplementary Figure 2 Follicular area is not significantly altered in the immunosuppressive phase of sepsis in *Fosl1* ^∆Mx^ mice compared to littermates. Representative H&E microscopy images and quantification of follicular area in the spleen of *Fosl1* ^∆Mx^ and the littermate mice after once (naïve) or twice (tolerant) stimulation of LPS. Scale bar = 200μm. Graph points indicate individual mice. Data are shown as mean ± s.e.m. *p < 0.05 by one-way ANOVA with Sidak’s multiple comparisons test.

Supplementary Figure 3 Uncropped blots results of (**Figure 3B**) Western blot detection of FRA-1 protein after LPS stimulation in littermate control and FRA-1 deficient BMDM in both naïve and tolerant state. GAPDH was used as loading control.

Supplementary Table 1 Primers sequences used for Real-Time PCR and ChIP-qPCR.
