# Supplementary figures and images for: The Transcription Factor FRA-1/AP-1 Controls Lipocalin-2 Expression and Inflammation in Sepsis Model
Source: Front Immunol. 2021 Oct 12;12:701675. doi: 10.3389/fimmu.2021.701675 (PMC8546226; doi:10.3389/fimmu.2021.701675)

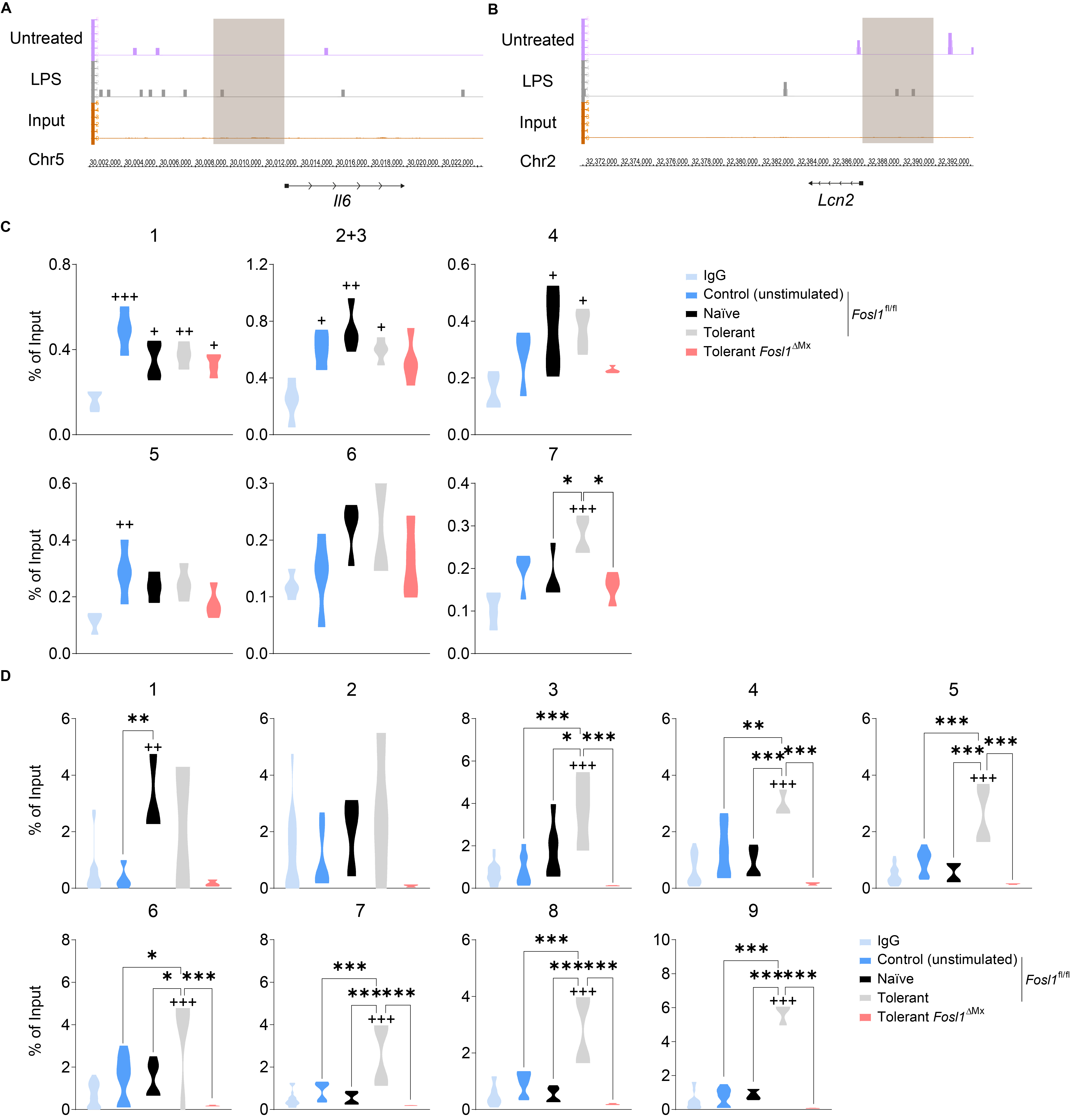

Supplement: Supplementary Figure 1 — FRA-1 binds to the promoters of Il6 and Lcn2. (A, B) ChIP-seq data (13) of thioglycollate-elicited macrophages from wild type mice, unstimulated or stimulated with LPS were used to identify FRA-1 target genes. Bindings of FRA-1 on the Il6 (A) and Lcn2 (B) promoter are indicated (C, D) ChIP assays showing the recruitment of the endogenous FRA-1 on the AP-1 binding sequence of Il6 (C) and Lcn2 (D) promoters in thioglycollate-elicited macrophages isolated from Fosl1 ΔMx or littermate mice after once (naïve) or twice (tolerant) stimulation of LPS. Data are shown as mean ± s.e.m. *p < 0.05; **p < 0.01; ***p < 0.001 by one-way ANOVA with Tukey’s multiple comparisons test. [file Image_1.jpeg]

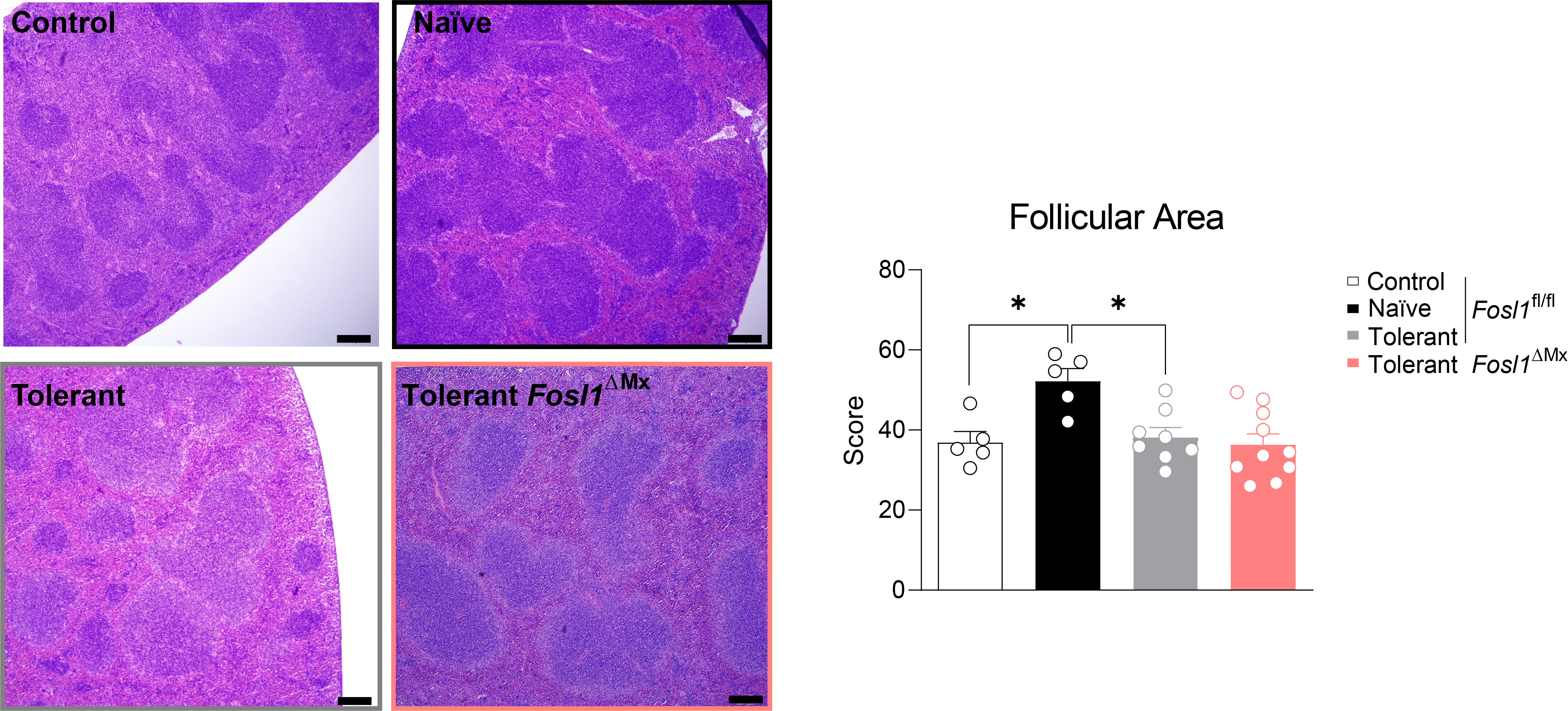

Supplement: Supplementary Figure 2 — Follicular area is not significantly altered in the immunosuppressive phase of sepsis in Fosl1 ∆Mx mice compared to littermates. Representative H&E microscopy images and quantification of follicular area in the spleen of Fosl1 ∆Mx and the littermate mice after once (naïve) or twice (tolerant) stimulation of LPS. Scale bar = 200μm. Graph points indicate individual mice. Data are shown as mean ± s.e.m. *p < 0.05 by one-way ANOVA with Sidak’s multiple comparisons test. [file Image_2.jpeg]

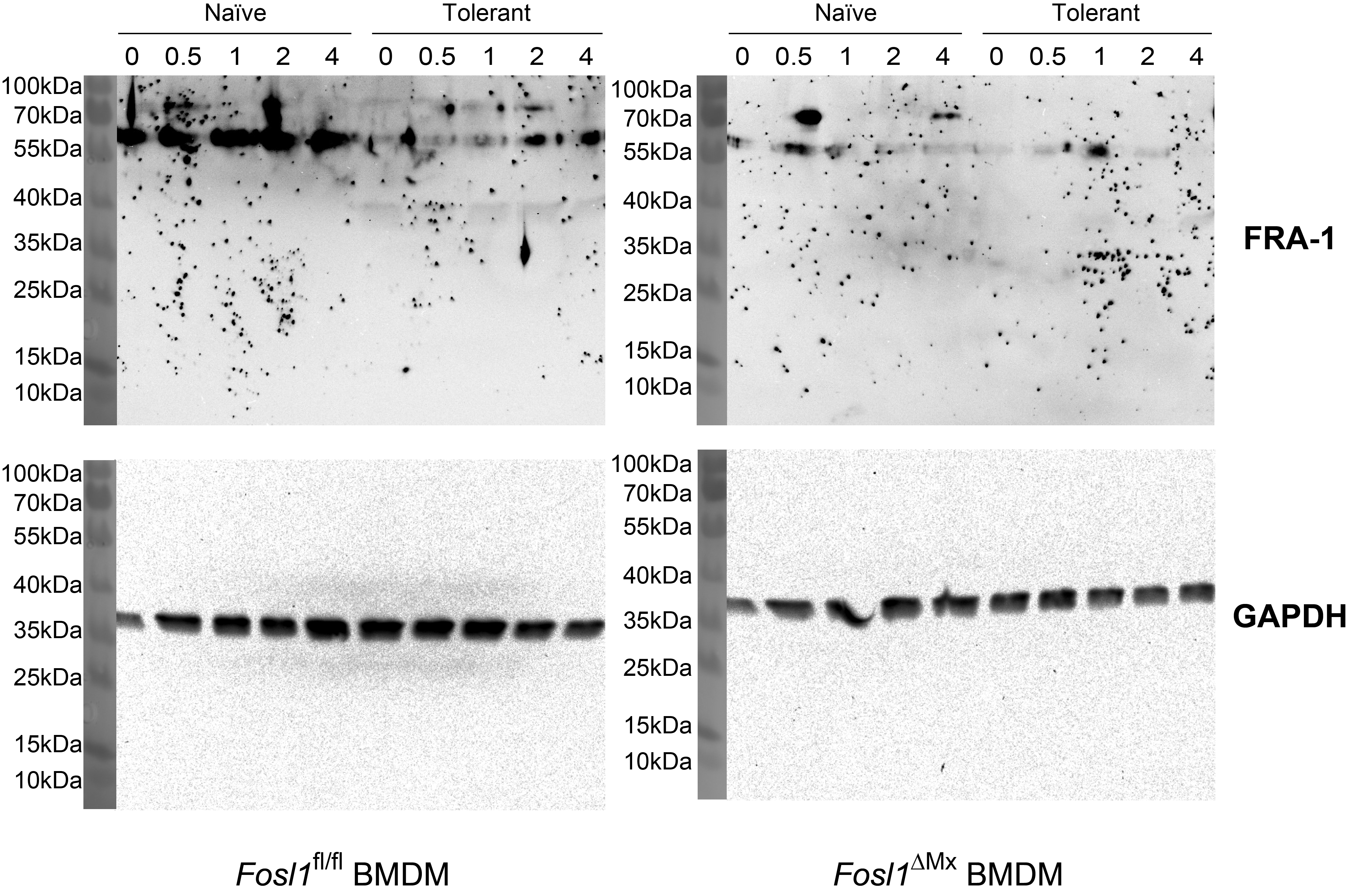

Supplement: Supplementary Figure 3 — Uncropped blots results of ( Figure 3B ) Western blot detection of FRA-1 protein after LPS stimulation in littermate control and FRA-1 deficient BMDM in both naïve and tolerant state. GAPDH was used as loading control. [file Image_3.jpeg]
